# Development of an online authentic radiology viewing and reporting platform to test the skills of radiology trainees in Low- and Middle-Income Countries

**DOI:** 10.1186/s12909-024-05899-w

**Published:** 2024-09-05

**Authors:** Hubert Vesselle, Justy Antony Chiramal, Stephen E. Hawes, Eric Schulze, Tham Nguyen, Rose Ndumia, Sudhir Vinayak

**Affiliations:** 1 Department of Radiology, Director of Global Health, University of Washington, 1959 N.E. Pacific Street, Box 357115, Seattle, WA 98195 USA; 2 Department of Epidemiology, University of Washington, Seattle, WA USA; 3 Lifetrack Medical Systems, Singapore, Singapore; 4 Independent Researcher, Can Tho, Vietnam; 5 Department of Radiology, Aga Khan University Hospital, Nairobi, Kenya

**Keywords:** Medical imaging education, Post-graduate physician remote testing, Authentic radiology practice evaluation, Novel scoring methods, Identification of educational needs, LMICs

## Abstract

**Background:**

Diagnostic radiology residents in low- and middle-income countries (LMICs) may have to provide significant contributions to the clinical workload before the completion of their residency training. Because of time constraints inherent to the delivery of acute care, some of the most clinically impactful diagnostic radiology errors arise from the use of Computed Tomography (CT) in the management of acutely ill patients. As a result, it is paramount to ensure that radiology trainees reach adequate skill levels prior to assuming independent on-call responsibilities. We partnered with the radiology residency program at the Aga Khan University Hospital in Nairobi (Kenya) to evaluate a novel cloud-based testing method that provides an authentic radiology viewing and interpretation environment. It is based on Lifetrack, a unique Google Chrome-based Picture Archiving and Communication System, that enables a complete viewing environment for any scan, and provides a novel report generation tool based on Active Templates which are a patented structured reporting method. We applied it to evaluate the skills of AKUHN trainees on entire CT scans representing the spectrum of acute non-trauma abdominal pathology encountered in a typical on-call setting. We aimed to demonstrate the feasibility of remotely testing the authentic practice of radiology and to show that important observations can be made from such a Lifetrack-based testing approach regarding the radiology skills of an individual practitioner or of a cohort of trainees.

**Methods:**

A total of 13 anonymized trainees with experience from 12 months to over 4 years took part in the study. Individually accessing the Lifetrack tool they were tested on 37 abdominal CT scans (including one normal scan) over six 2-hour sessions on consecutive days. All cases carried the same clinical history of acute abdominal pain. During each session the trainees accessed the corresponding Lifetrack test set using clinical workstations, reviewed the CT scans, and formulated an opinion for the acute diagnosis, any secondary pathology, and incidental findings on the scan. Their scan interpretations were composed using the Lifetrack report generation system based on active templates in which segments of text can be selected to assemble a detailed report. All reports generated by the trainees were scored on four different interpretive components: (a) acute diagnosis, (b) unrelated secondary diagnosis, (c) number of missed incidental findings, and (d) number of overcalls. A 3-score aggregate was defined from the first three interpretive elements. A cumulative score modified the 3-score aggregate for the negative effect of interpretive overcalls.

**Results:**

A total of 436 scan interpretations and scores were available from 13 trainees tested on 37 cases. The acute diagnosis score ranged from 0 to 1 with a mean of 0.68 ± 0.36 and median of 0.78 (IQR: 0.5-1), and there were 436 scores. An unrelated secondary diagnosis was present in 11 cases, resulting in 130 secondary diagnosis scores. The unrelated secondary diagnosis score ranged from 0 to 1, with mean score of 0.48 ± 0.46 and median of 0.5 (IQR: 0–1). There were 32 cases with incidental findings, yielding 390 scores for incidental findings. The number of missed incidental findings ranged from 0 to 5 with a median at 1 (IQR: 1–2). The incidental findings score ranged from 0 to 1 with a mean of 0.4 ± 0.38 and median of 0.33 (IQR: 0- 0.66). The number of overcalls ranged from 0 to 3 with a median at 0 (IQR: 0–1) and a mean of 0.36 ± 0.63. The 3-score aggregate ranged from 0 to 100 with a mean of 65.5 ± 32.5 and median of 77.3 (IQR: 45.0, 92.5). The cumulative score ranged from − 30 to 100 with a mean of 61.9 ± 35.5 and median of 71.4 (IQR: 37.4, 92.0). The mean acute diagnosis scores and SD by training period were 0.62 ± 0.03, 0.80 ± 0.05, 0.71 ± 0.05, 0.58 ± 0.07, and 0.66 ± 0.05 for trainees with ≤ 12 months, 12–24 months, 24–36 months, 36–48 months and > 48 months respectively. The mean acute diagnosis score of 12–24 months training was the only statistically significant greater score when compared to ≤ 12 months by the ANOVA with Tukey testing (*p* = 0.0002). We found a similar trend with distribution of 3-score aggregates and cumulative scores. There were no significant associations when the training period was categorized as less than and more than 2 years. We looked at the distribution of the 3-score aggregate versus the number of overcalls by trainee, and we found that the 3-score aggregate was inversely related to the number of overcalls. Heatmaps and raincloud plots provided an illustrative means to visualize the relative performance of trainees across cases.

**Conclusion:**

We demonstrated the feasibility of remotely testing the authentic practice of radiology and showed that important observations can be made from our Lifetrack-based testing approach regarding radiology skills of an individual or a cohort. From observed weaknesses areas for targeted teaching can be implemented, and retesting could reveal their impact. This methodology can be customized to different LMIC environments and expanded to board certification examinations.

## Introduction

Low- and middle-income countries (LMICs) are now equipping their hospitals with state-of-the-art medical imaging equipment (Ultrasound, CT, MRI, and nuclear medicine scanners) and the use of diagnostic imaging is steadily increasing across many countries. However, the clinical impact of increased imaging utilization is hampered by the insufficient number of well-trained radiologists to make optimal use of such modern imaging technology in order to enhance the lives of patients through accurate diagnosis and monitoring of disease response to therapy. Increasing the supply of diagnostic radiologists in LMICs will require increasing training programs’ capacity but also enhancing the quality of education across all radiology subspecialties. Contrary to their counterparts in high-income countries (HICs), radiology training programs in LMICs have a limited number of teaching faculty or may lack faculty with sub-specialty expertise in certain specific areas of radiology. Hence there is a need for global health radiology efforts towards education [1–4]. Trainees in LMICs may have to provide significant contributions to the clinical workload before the completion of their residency training. Because of time constraints inherent to the delivery of acute care, some of the most clinically impactful diagnostic radiology errors arise from the use of Computed Tomography (CT) in the management of acutely ill patients either presenting to an emergency room or already hospitalized. As a result, it is paramount to ensure that radiology trainees reach adequate skill levels prior to assuming independent on-call responsibilities.

Objective structured clinical/practical examinations (OSCE/OSPE) have been widely used in medical education with simulated patients or laboratory data since their introduction by Harden [5]. OSCE/OSPE are designed to test trainees on preset clinical scenarios with many clinical inputs, and have successfully integrated a single chest radiograph, or a few curated images for the purpose of evaluating medical students or radiography technologists [6–8]. However, OSCE/OSPE do not lend themselves to the evaluation of the actual practice of cross-sectional imaging (e.g.: formal CT or MRI scan interpretations) where a single clinical scan is made of hundreds of images that need to be thoroughly searched for abnormalities, then analyzed together to develop a final acute diagnosis. An OSCE approach based on a few curated images and focused questions would circumvent the searching task required of radiologists facing a clinical CT scan and would not allow for the generation of a detailed radiology report with identification of a possible secondary diagnosis, incidental findings, and possible errors in search and characterization leading to the overcalling of normal findings.

We partnered with the radiology residency training program at the Aga Khan University Hospital in Nairobi, Kenya (AKUHN) to evaluate a novel cloud-based testing method that provides an authentic radiology viewing and interpretation environment for practicing radiologists and radiology trainees. An authentic radiology environment is one that enables the clinical review of any large diagnostic image set and the generation of a comprehensive clinical report. Our testing method is based on Lifetrack (Lifetrack Medical Systems, Singapore), a unique and patented Google Chrome-based Picture Archiving and Communication System (PACS), that enables a complete viewing environment for any scan, even one with several hundred images and which provides a novel report generation tool based on active templates. We applied this new technology to evaluate the skills of AKUHN trainees using complete CT exams selected to represent the spectrum of acute non-trauma abdominal pathology encountered in a typical on-call setting. The trainees reviewed these clinical scans on workstations in this authentic Lifetrack environment and generated their scan interpretations using templated reports, thereby enabling an objective evaluation of their diagnostic radiology skills over a wide range of representative acute abdominal pathologies. It was our goal to demonstrate the feasibility of remotely testing the authentic practice of radiology and to show that important observations can be made from such a Lifetrack-based testing approach regarding the radiology skills of an individual practitioner or of a cohort of trainees.

## Methods

### Setting

AKUHN is a 289-bed hospital in Nairobi, Kenya. It is a recognized center of radiology excellence for the horn of Africa being the first hospital in the region to receive the coveted Joint Commission International Accreditation (JCIA). It is home to Radiology 14 faculty members (1 Associate and 6 Assistant Professors, 7 senior instructors) as well as 6 instructors and a part-time physicist. To be designated as faculty member, a practitioner must have completed radiology residency training, have board recognition, and fellowship training. The radiology residency at AKUHN is a structured four-year program which enrolls up to 4 trainees every year. The radiology program is intensive with multiple diagnostic and interventional radiology scans and procedures performed every day. An average of 82,000 radiology examinations are conducted yearly at AKUHN. The radiology department has state-of-the-art imaging equipment including digital X-ray and fluoroscopy units, a digital mammography system with tomosynthesis, seven ultrasonography units, a 64-slice MDCT, a cone beam CT, 1.5T and 3T MRI scanners, a SPECT/CT and a PET/CT scanner with an on-site cyclotron, and an angiography suite.

Our clinical skills study was conducted in December 2020. It was approved by the Aga Khan University, Nairobi Institutional Ethics Review Committee. All study participants gave written informed consent before participating in this study. The test takers were anonymized and given access to the Lifetrack tool which tested them on 37 abdominal CT scans over six 2-hour sessions. This was conducted between 7-9AM EAT in the AKUHN Radiology department with supervision by the residency program director (RN). The subjects in our study had undergone varying lengths of radiology training reflecting their time in the residency program which was recorded in a pre-test questionnaire embedded in the first testing session. All the data was collected and recorded in Microsoft Excel spreadsheets. After the test was concluded, all 37 cases were reviewed with the trainees by one of the authors (HV) during weekly online teaching sessions to maximize the educational benefit from each case.

### Lifetrack

**Fig. 1:**
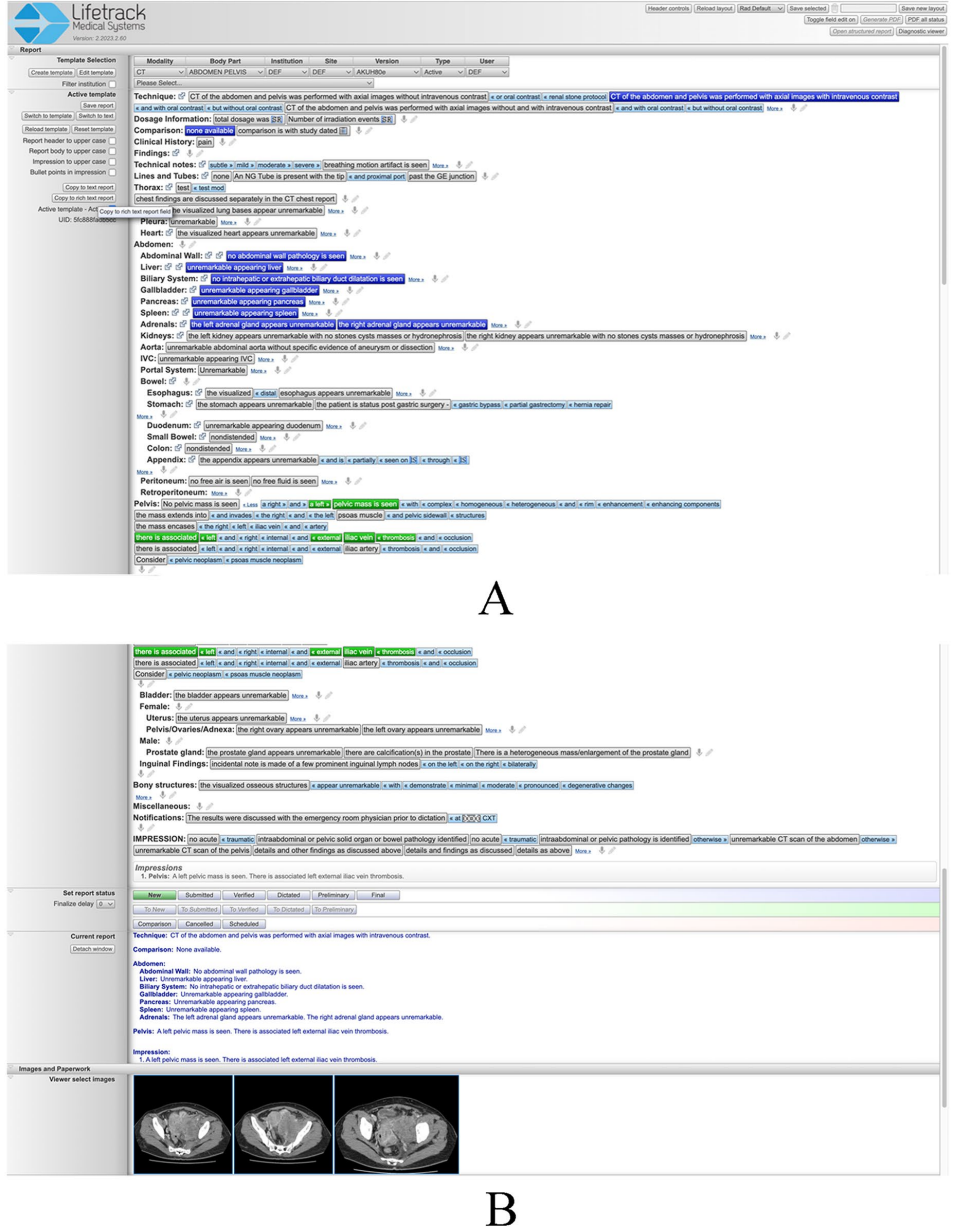
**A**& **B**: These two images capture an entire example report composed using the Lifetrack active report template for abdomen and pelvis CT examination. The template is selected at the top from a drop-down menu. The selectable elements of text are shown as tabs that can be pressed to constitute entire sentences identifiable in the current report visible at the bottom of Fig. 1B. If pressed once (blue highlight) the text in a tab will be displayed in the body of the report only. If pressed twice (green highlight), the text will be present in both the body and impression of the final report as in this case for the pelvic mass and venous thrombosis findings. An additional feature of the Lifetrack active report template is the ability to insert relevant images in the report as displayed in Fig. 1B

Lifetrack is an FDA-approved next generation distributed Radiology Information System (RIS)/PACS architecture produced by Lifetrack Medical Systems (https://www.lifetrackmed.com/). Lifetrack incorporates integrated decision support features and automatic feedback loops which enable efficient quality diagnosis by radiologists. The software is also used for radiologist training and capacity building in those countries where there is a scarcity of radiologists. The Lifetrack PACS System has a patented Active Template technology which is used for efficient reading of scans by practicing radiologists (Fig. 1A and B). It can also be used as a training system since the Active Templates allow each plain English sentence template block to be encoded with data such that any interpretive choice made by the radiology trainee while building a report can be turned into an automatically scorable “answer” in an Excel spreadsheet. Prior to the start of our study, a separate two-hour orientation session reproducing the exact testing environment was organized. It allowed for study participants to navigate as many example CT scans as necessary for them to become comfortable using the Lifetrack image viewer and to learn to generate reports by selecting segments of text as illustrated in Fig. 1A and B.

### Selection of CT scans for the study

The list of pathologies for the examination was selected by the principal investigator (HV) and a junior radiology colleague (TN), from a textbook on emergency diagnostic radiology that encompasses the spectrum of diagnoses for an acute abdomen [9]. In order to maintain the set of CT cases to a manageable number while providing a representative sample of pathologies, only non-trauma abdominal and pelvic diagnoses were selected. CT scans of the abdomen and pelvis for the selected pathologies were identified from the Lifetrack server of clinically-documented anonymized teaching cases. A total of 36 cases of acute abdominal pathology were curated for the purpose of this CT interpretation skill evaluation. An additional normal CT scan of the abdomen and pelvis was included. Formal interpretations of the 37 scans, with respect to acute diagnosis and its CT imaging features, any secondary non-acute but concurrent pathology, and any incidental findings, were recorded by the authors (HV, TN) for subsequent scoring of trainees’ scan interpretations. All cases were organized in 5 sets of 6 cases and one set of 7 cases (containing the normal CT scan) loaded as 6 separate test sessions accessible on the teaching server of Lifetrack. All scans were of de novo pathologies so that neither prior scan comparisons nor additional clinical information (beyond acute abdominal pain) was necessary to perform an accurate scan interpretation.

In selecting the cases for the test (Table 1), care was taken to identify examples that were representative of the spectrum of presentations for the chosen pathologies and that the scans were performed using good imaging technique. Eleven of them included an important second non-acute diagnosis that trainees were expected to identify, characterize, and report. Incidental findings were wide-ranging as reported in Table 1.

**Table 1:** Description of abdominal CT cases with their acute diagnosis, unrelated secondary diagnosis, and incidental findings

Case Number	Acute diagnosis (*n* = 37)	Unrelated secondary diagnosis (*n* = 11)	Incidental findings (*n* = 32)
1.	Sigmoid volvulus with closed-loop obstruction	None	Ascites; small right pleural effusion.
2.	Left pelvic mass with left ureteral obstruction; left external iliac vein DVT.	Retroperitoneal lymph nodes	None
3.	Diverticulitis with perforation and abscesses	None	Bilateral small pleural effusions
4.	Small bowel obstruction due to a right sciatic foramen hernia	Right middle lobe traction bronchiectasis; Right lung lower lobe nodular consolidation	DJD; duodenal diverticulum; free fluid.
5.	Sigmoid diverticulitis with microperforation	None	Gallstones; s/p appendectomy.
6.	Interstitial pancreatitis with pseudocysts and ascites	None	Bilateral pleural effusions; ovarian cysts; mild biliary dilatation.
7.	Small bowel obstruction with lead point	None	Gastric distension; abdominal incision with air; free fluid; bilateral renal cysts.
8.	Thrombosis of superior mesenteric vein and right portal vein.	Postpartum uterus	Differential attenuation in left hepatic lobe
9.	Duodenal hematoma and gastric distension	None	Trace free pelvic fluid
10.	Pyelonephritis with renal abscess	None	Atelectasis at left lung base; small associated left retroperitoneal nodes; trace of pelvic free fluid.
11.	Splenomegaly and old splenic infarcts.	Cirrhosis; portal hypertension with large portosystemic collateral.	Non-obstructive left kidney stone; subtle increased attenuation in renal medulla bilaterally; left renal hypodensity.
12.	Obstructing stone at the left UVJ with hydronephrosis, hydroureter and perinephric fluid.	Atrophic right kidney	Two tiny lung nodules at each lung base; right renal hypodensity; IUD in place; bilateral L5-S1 pars defects with grade 1 anterolisthesis.
13.	Right renal artery occlusion	None	Small accessory renal artery perfusing part of right renal upper pole.
14.	Ruptured ectopic pregnancy (right adnexal mass and blood in peritoneal cavity)	Autosomal Dominant Polycystic Kidney Disease	None
15.	Leaking Abdominal Aortic Aneurysm	Misplaced NG tube in distal esophagus	None
16.	Splenic lesion with daughter cysts - Hydatid disease	None	Malpositioned IUD in uterus; calcified granuloma in right gluteal region.
17.	Bilateral tubo-ovarian abscesses	None	Reactive retroperitoneal and pelvic nodes
18.	Acute on chronic pancreatitis (calcifications, pancreatic duct dilatation, pseudocysts)	None	Renal cysts
19.	Mesenteric adenitis	None	Trace of free pelvic fluid
20.	Acute appendicitis without perforation	Atrophic right kidney.	Two non-obstructing left renal stones
21.	Colitis	None	Small hepatic cyst; focal fat next to falciform ligament; uterine fibroids; free pelvic fluid; small RLQ nodes.
22.	Abdominal aortic aneurysm with small dissection and mural thrombus	S/p anterior abdominal wall hernia repair; new bowel containing hernia caudal to the repair; no bowel strangulation.	Severe degenerative disc disease of the spine; small seroma in the anterior abdominal wall; mild cirrhosis.
23.	Terminal ileitis	None	None
24.	Pyelonephritis (striated left nephrogram)	None	Splenules; trace free pelvic fluid.
25.	Epiploic appendagitis	None	Focal fat next to falciform ligament
26.	Acute cholecystitis	None	Non-obstructive left renal stone
27.	Thrombus in portal veins and SMV extending into small bowel branches. Small bowel ischemia due to venous thrombosis. Splenic infarct with small clot in distal splenic vein. Free perihepatic fluid.	None	Bilateral small renal cysts; non-enlarged retroperitoneal nodes; spina bifida occulta at L5 and S1.
28.	Acute pancreatitis with peripancreatic fluid and early pseudocyst formation.	None	Emphysema at left lung base; post cholecystectomy status; indeterminate liver hypodensity; biliary stents in CBD
29.	Ruptured appendix with free air and free localized RLQ fluid. Enlarged right lower quadrant lymph nodes. Free intraperitoneal fluid. RLQ fat stranding. Appendicoliths.	None	Focal fat next to falciform ligament; mild delay of right nephrogram and prominent renal pelvis due to spasm of the right ureter coursing along the site of RLQ infection; retroaortic left renal vein.
30.	Hydatid liver abscesses crossing the chest wall, with peritoneal abscesses.	Right lower lobe and right middle lobe tree-in-bud opacities, likely TB.	Bilateral small renal cysts; liver cysts.
31.	Right lower quadrant bowel perforation from fish bone; free air.	None	Renal scars; gallstones; fatty infiltration of the liver; small infra-renal abdominal aortic aneurysm.
32.	None (normal CT scan of the abdomen and pelvis)	None	Two healed right lower rib fractures; non-obstructive bilateral renal calculi.
33.	Perinephric hemorrhage from a left renal angiomyolipoma	None	Tiny scattered liver hypodensities; focal fat adjacent to the falciform ligament; trace of free fluid in the pelvis; uterine fibroids; jejunal thickening.
34.	Ileus	None	Simple hepatic cyst; free pelvic fluid.
35.	Swallowed toothpick causing stomach wall abscess and liver abscess; middle hepatic vein thrombosis.	None	Right renal cyst; small gallbladder stone.
36.	Right psoas muscle abscess with adjacent L4-5 spondylodiscitis.	Thrombus in IVC, left common iliac vein and left renal vein/gonadal vein	Hepatic hypodensity; renal cysts; plate atelectasis at lung bases; healing fracture of left 8th rib.
37.	Pancreatic cancer (1) with biliary dilatation (2); pancreatic duct dilatation (3); pancreatic tail atrophy (4); invasion of several arteries and veins (5). Porto-venous collaterals (6).	None	None

*Abbreviations*: DJD: degenerative joint disease; DVT: deep vein thrombosis; TB: tuberculosis; UVJ: ureterovesical junction; s/p: status-post; IUD: intrauterine device; NG: nasogastric; RLQ: right lower quadrant; CBD: common bile duct; GB: gallbladder; IVC: inferior vena cava; fx: fracture

The 37 scans were presented to the trainees in 6 sessions of two hours each, on 6 consecutive days. All cases carried the same clinical history of acute abdominal pain which was provided to the trainees. During each session the trainees accessed the corresponding Lifetrack test set using their reading workstations in the AKUHN Radiology department and loaded the cases. They reviewed the CT scans with the Lifetrack viewing tools and formulated an opinion with respect to the acute diagnosis depicted on each CT scan as well as any other concurrent pathology that may be present on the scan. This process was identical to the clinical viewing and interpretation environment deployed in any radiology reading room. Trainees entered their interpretation of each scan by composing a formal radiology report using the patented Lifetrack report generation system based on active templates in which segments of text can be selected to formulate a report (see example in Fig. 1A and B). This reporting system is designed to be exhaustive and provides the ability to create any report including typing or dictating free text if needed. The final report and its impression are a record of a trainee’s opinion of the primary acute abdominal diagnosis and may have included a secondary non-acute diagnosis (if present on the scan), and incidental findings as in any practicing radiologist’s report. All trainees were aware that their scan interpretations would be graded as part of this study and that for each scan they were expected to identify the acute diagnosis, any unrelated secondary diagnosis, incidental findings, and to avoid overcalling normal findings as pathology.

Each trainee was identified by a unique coded identifier that maintained their anonymity and enabled all of the trainee’s scan interpretations to be pooled across all six testing sessions. Each test CT scan was also labeled with a unique identifier for tracking of results and pooling of trainee performance for each case and pathology. A total of 13 trainees took part in the study all ranging in experience from 12 months to more than 4 years (three of the subjects had recently graduated from the residency training program).

### Scores and scoring system

All reports generated by the trainees were reviewed and scored by a senior radiologist specialized in cross-sectional imaging (HV) based on a diagnostic consensus from the co-authors (HV, TN). Every case had four different interpretive components that each trainee was scored on: (a) acute diagnosis, (b) unrelated secondary diagnosis, (c) number of missed incidental findings, and (d) number of overcalls.

The acute/primary diagnosis score ranged from 0 (no credit) to 1 (full credit) based on how well the trainee identified the primary diagnosis, i.e. the main cause of abdominal pain the patient presented with. The patient’s urgent management depends on that diagnosis. Partial credit was given for identifying the scan abnormalities associated with this primary diagnosis. If this acute diagnosis contained several key imaging features on the CT scan, incremental credit was given if the trainee identified more of these features: for example, if the primary diagnosis had 4 imaging features and the trainee only identified 3 of them, they received a 0.75 credit for the acute/primary diagnosis. If the primary lesion/abnormality was identified but the correct diagnosis was only mentioned as part of a differential diagnostic list (DDx), an additional partial credit was given for the latter but inversely proportional to how many diagnoses were listed in the DDx. For example, in the third session (case 16), a trainee identified the splenic lesion and provided a DDx of abscess versus metastasis. Therefore 0.5 credit was granted for identifying the CT abnormality, but only 0.5/2 = 0.25 extra credit was earned for mentioning the correct diagnosis as part of a DDx, for a total score of 0.5 + 0.25 = 0.75. In contrast, a trainee who identified the splenic lesion but failed to characterize it (and did not provide a DDx that included the correct diagnosis), received an acute diagnosis score of 0.5 only. The significance of the acute diagnosis score is that it reveals the ability and thoroughness of the trainee to diagnose acute abdominal disease that they are likely to confront during overnight call duties and for which lack of correct diagnosis can have deleterious effects on patient care.

The unrelated secondary diagnosis score ranged from 0 to 1 and refers to an important other diagnosis that the trainee needs to make based on the CT scan provided but one that is not related to the primary diagnosis causing the acute abdominal pain. This unrelated secondary diagnosis would warrant further evaluation on a non-emergent basis. Trainees could get a partial score based on the answer provided. The significance of this secondary diagnosis score is that it tests the thoroughness of the trainee in identifying all medically important features of a CT scan and not stop at the acute diagnosis once established. It also addresses the ability of a trainee to distinguish medically important but non-urgent pathology from acutely urgent pathology.

Incidental findings are imaging findings on the CT scan that do not carry an urgent or medically important significance but that are meant to be identified and reported as they could be misinterpreted. They reveal the visual detection ability and the thoroughness of the trainee and their ability to not over diagnose benign incidental findings. The incidental findings score ranged from 0 to 1. It was scored as the percentage of the incidental findings that the trainee identified. A trainee received full credit of 1 for missing no incidental finding and the score was proportionately reduced depending on how many of the incidental findings they missed. For those CT cases without incidental finding no incidental finding score was recorded.

Number of overcalls could range from 0 to greater than 1 based on the number of overcalls that the trainee had made. An overcall is a wrong diagnosis or wrong interpretation of a scan finding. Trainees may also mis-interpret normal findings or normal variants as pathology leading to a wrong clinical recommendation being made, and to wrong treatment. This was inversely scored, so that the trainee received full credit for making no overcall. However, the cumulative score (see below) was reduced for every overcall made. The significance of this measure is that overcalls can be a sign of “interpretive insecurity” or incomplete/poor training, in that a trainee unsure of themselves may provide multiple diagnoses for a scan in an effort to catch the true diagnosis in a broad list of possibilities.

### 3-score aggregate and cumulative score

We developed a 3-score aggregate that was the weighted sum of the three scores: Acute diagnosis score, unrelated secondary diagnosis score, and score for incidental findings with weights of 80%, 10%, and 10% respectively. Thus the 3-score aggregate could range from 0 to 100. Since the acute diagnosis is the key interpretation finding that we expect an on-call trainee to diagnose promptly in order to accurately guide patient management, we gave the acute diagnosis score an 80% weight in the 3-score aggregate. The unrelated secondary diagnosis and missed incidental findings were given a 10% weight each, since those were findings that were still relevant in evaluating a trainee’s skills but not as significant in the acute care setting. In cases where there was no secondary diagnosis or no incidental finding the corresponding 10% weight was added to the acute diagnosis score so that the maximum possible score remained 100.

$$\eqalign{&{\bf{3\text{-}score}}\,{\rm{ }}{\bf{aggregate}}{\rm{ }} \cr &\quad = \,{\bf{80}}\,*\,{\bf{Acute}}\,{\rm{ }}\,{\bf{diagnosis}}{\rm{ }}\,{\bf{score}}{\rm{ }} \cr & \quad+ \,{\bf{10}}\,*\,{\bf{Unrelated}}\,{\rm{ }}{\bf{secondary}}{\rm{ }}\,{\bf{diagnosis}}{\rm{ }}\,{\bf{score}}{\rm{ }} \cr & \quad+\,{\bf{10}}\,*\,{\bf{Score}}\,{\rm{ }}{\bf\,{for}}{\rm{ }}\,{\bf{incidental}}{\rm{ }}\,{\bf{findings}} \cr}$$

Similarly, we also developed a cumulative score to account for all 4 components of the interpretation of an acute abdominal CT scan: acute diagnosis, unrelated secondary diagnosis, incidental findings, and overcalls. The cumulative score is defined as the 3-score aggregate (0-100 value) minus 10 for every overcall made by the trainee in the interpretation of the case. Thus, the cumulative score of a trainee on a case could range from a maximum of 100 down to negative values if many overcalls were made.

$$\eqalign{& {\bf{Cumulative}}\,{\rm{ }}{\bf{score}}\, = \, \cr & {\bf{3}}\text{-}{\bf{score}}\,{\rm{ }}{\bf{aggregate}}\, - \,\left( {{\bf{10}}\,*\,{\bf{Number}}\,{\rm{ }}{\bf{of}}\,{\rm{ }}{\bf{overcalls}}} \right) \cr}$$

### Data analysis

We used descriptive statistics to summarize continuous variables as medians with interquartile ranges (IQRs) and mean +/- standard deviation (SD). We analyzed the data using R (R Foundation for Statistical Computing, Vienna, Austria) and developed plots and heatmaps using the R package ggplot2. We fit linear regression to analyze the association of acute diagnosis and cumulative scores with length of radiology training. We considered *p* < 0.05 as statistically significant for all analyses. We divided the length of training into five categories (≤ 12 months, 12–24 months, 24–36 months, 36–48 months, and > 48 months) and two categories (< 24 months, and ≥ 24 months), and compared the cumulative scores, 3-score aggregate, and acute diagnostic scores of trainees. Our hypothesis was that the scores would be higher as the length of training gets longer. Similarly, we fit a model to look at the association of the number of overcalls with the training period of trainees, with a hypothesis that the number of overcalls would decrease with increased training. We also looked at the distribution of 3-score aggregate versus the number of overcalls by trainees, assuming that the scores should be inversely proportional to the number of overcalls.

## Results

There were 14 radiology trainees who took part in this study. Trainee 11 (Tr11) was assigned a number but never participated in any of the testing sessions. Trainee 2 (Tr2) only attempted 7 cases out of a total of 37 cases because of a planned vacation, so we excluded this trainee for comparisons of scores between trainees, but we included their scores when reporting and analyzing results on a per case basis. There were two sessions when a trainee missed reporting on one case each, one session where a trainee missed 5 cases, and one of the trainees missed an entire testing session (7 cases) due to on-call duties on the night prior to a session. During the first session one of the trainees erroneously entered the report associated with another scan; we assumed this was a technical issue on the first day and excluded the corresponding score from our analysis. Thus, we had scan interpretations and scores available on a total of 436 cases.

Evaluating the 12 remaining trainees by the length of their radiology training, there were three trainees in each category who had completed at least 1 year, 2 years and 3 years of training. There was one trainee who had completed 4 years of training and two who had more than 4 years of training.

**Fig. 2:**
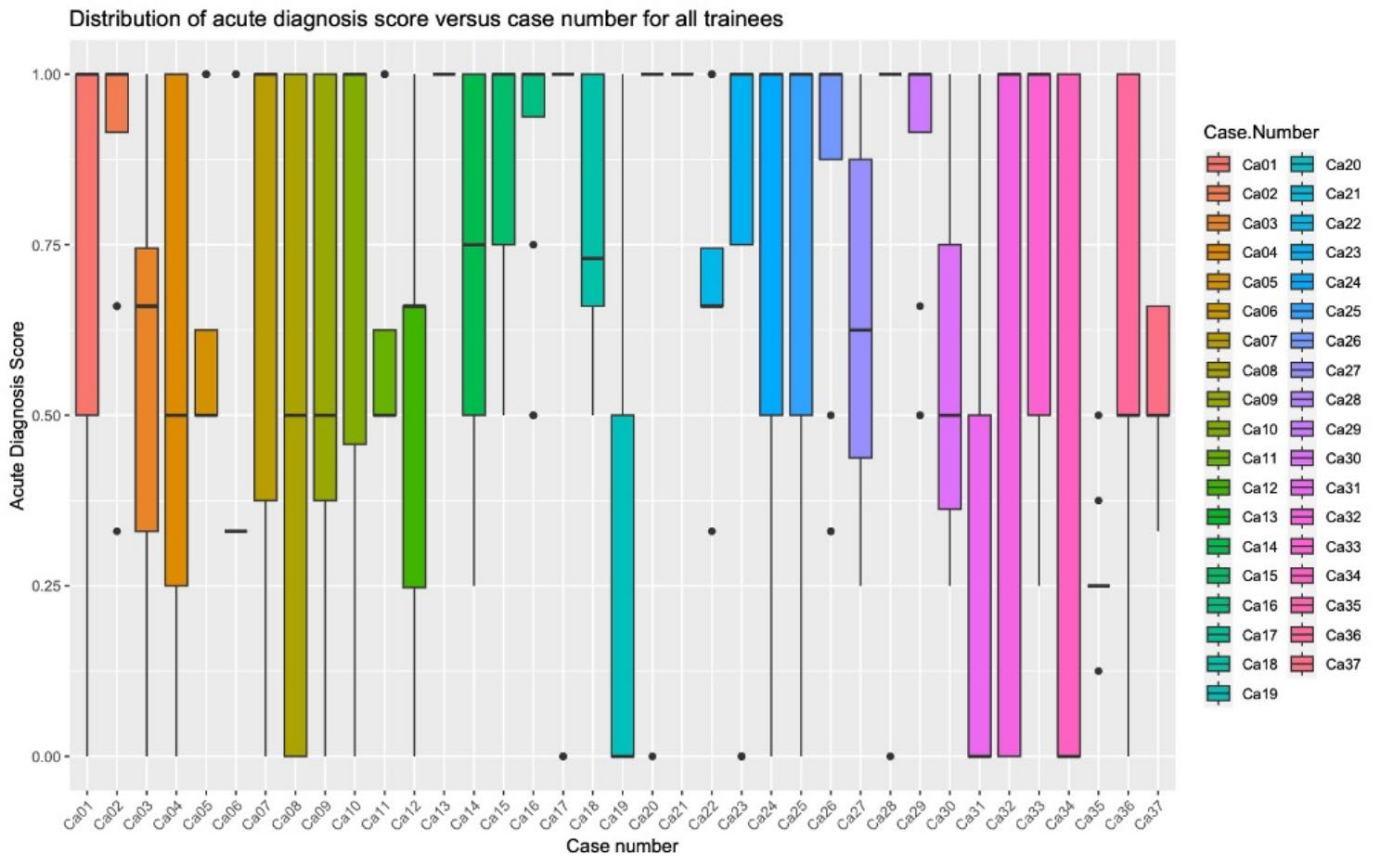
Boxplot distribution of the acute diagnosis score versus case number for all trainees

The acute diagnosis score ranged from 0 to 1 with a mean of 0.68 ± 0.36 and median of 0.78 (IQR: 0.5-1), and there were 436 scores (Fig. 2). An unrelated secondary diagnosis was present in 11 cases, resulting in 130 secondary diagnosis scores. The unrelated secondary diagnosis score ranged from 0 to 1, with mean score of 0.48 ± 0.46 and median of 0.5 (IQR: 0–1). There were 32 cases that had incidental findings, thus we had 390 scores for incidental findings. The number of missed incidental findings ranged from 0 to 5 with a median at 1 (IQR: 1–2). The incidental findings score ranged from 0 to 1 with a mean of 0.4 ± 0.38 and median of 0.33 (IQR: 0- 0.66). The number of overcalls ranged from 0 to 3 with a median at 0 (IQR: 0–1) and a mean of 0.36 ± 0.63.

### Scores by case

Across cases, we looked at the distribution of the acute diagnosis scores (shown in Fig. 2). The lowest mean acute diagnosis score was 0.27 ± 0.09 for case number Ca35 (diagnosis: Liver abscess; stomach wall abscess; middle hepatic vein thrombosis; toothpick in left lobe of liver). The highest mean acute diagnosis score was 100% for Ca13 (right renal artery occlusion) and Ca21 (colitis).

Other than Ca35, there were only 4 other cases where the mean acute diagnosis score was less than 50%: (a) Ca31: Fish bone perforation of bowel (mean score = 0.30 ± 0.37); (b) Ca19: Mesenteric adenitis (mean score = 0.32 ± 0.40); (c) Ca6: Interstitial pancreatitis with pseudocysts and ascites (mean score = 0.44 ± 0.26); and (d) Ca12: Obstructing stone at the left UVJ (mean score = 0.50 ± 0.33).

### 3-score aggregate

**Fig. 3:**
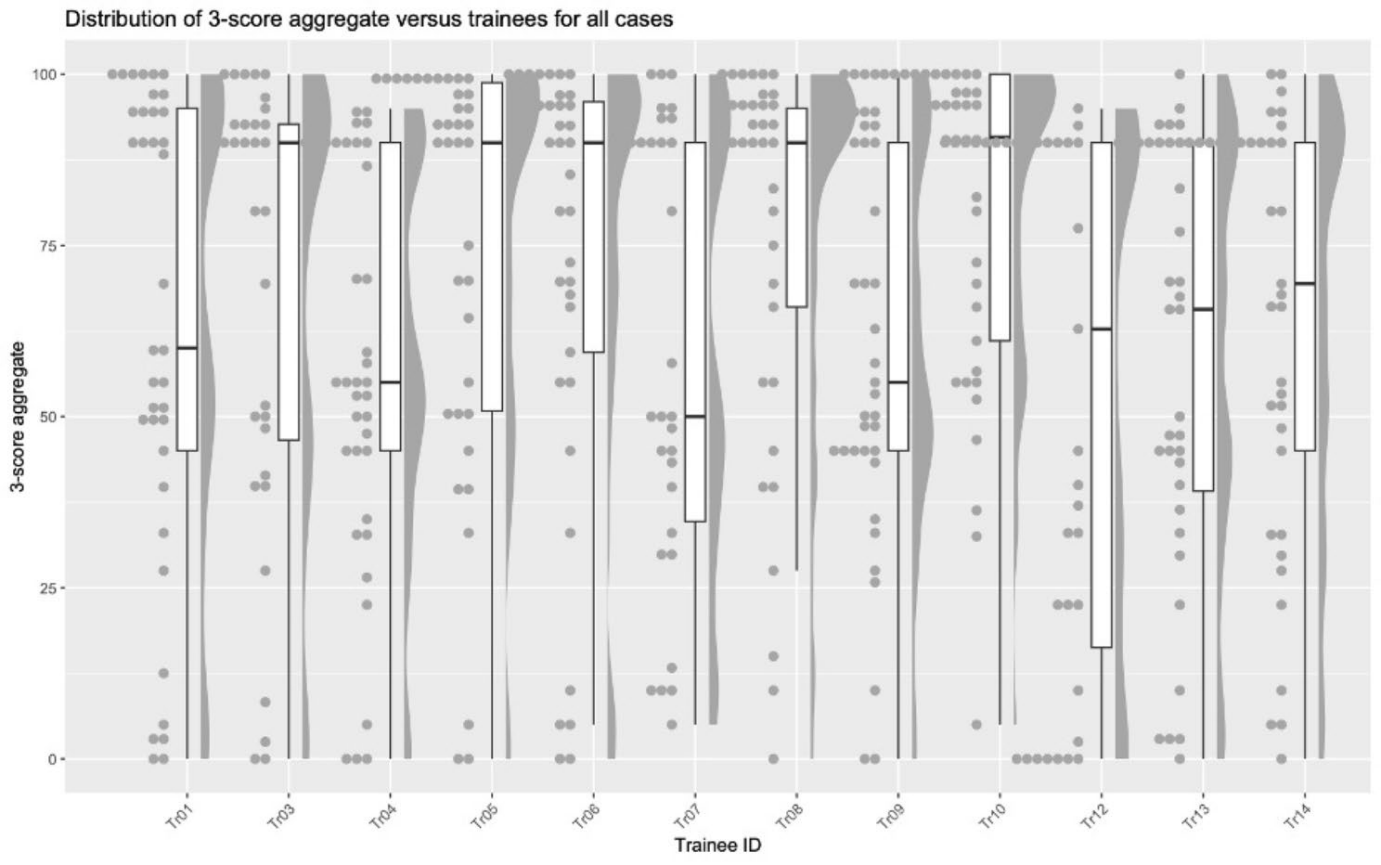
Raincloud plot showing the distribution of the 3-score aggregates and IQR by trainee

The 3-score aggregate for every case by trainee was calculated as described above. The distribution of these scores by case number is shown on Fig. 3. The 3-score aggregate ranged from 0 to 100 with a mean of 65.5 ± 32.5 and median of 77.3 (IQR: 45.0, 92.5).

**Fig. 4:**
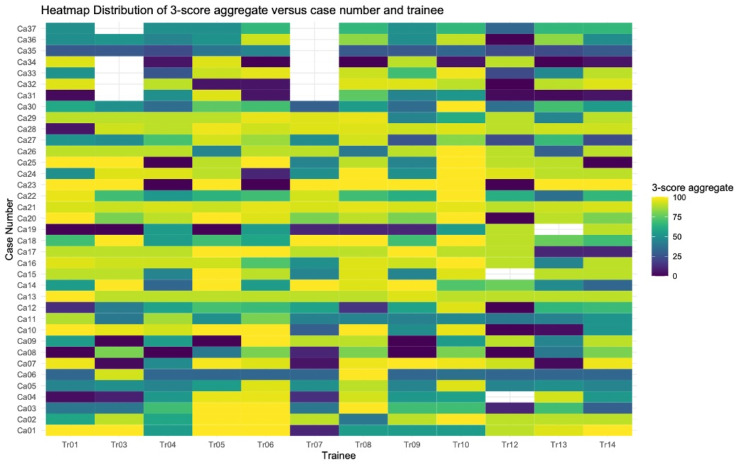
Heatmap distribution of the 3-score aggregate by case number and trainee

The heat map in Fig. 4. shows the distribution of 3-score aggregate for all trainees by individual case number. Shades of colors range from dark blue (0) to yellow (+ 100) depending on the 3-score aggregate. A white cell corresponds to a case not interpreted by a trainee.

### Cumulative scores

**Fig. 5:**
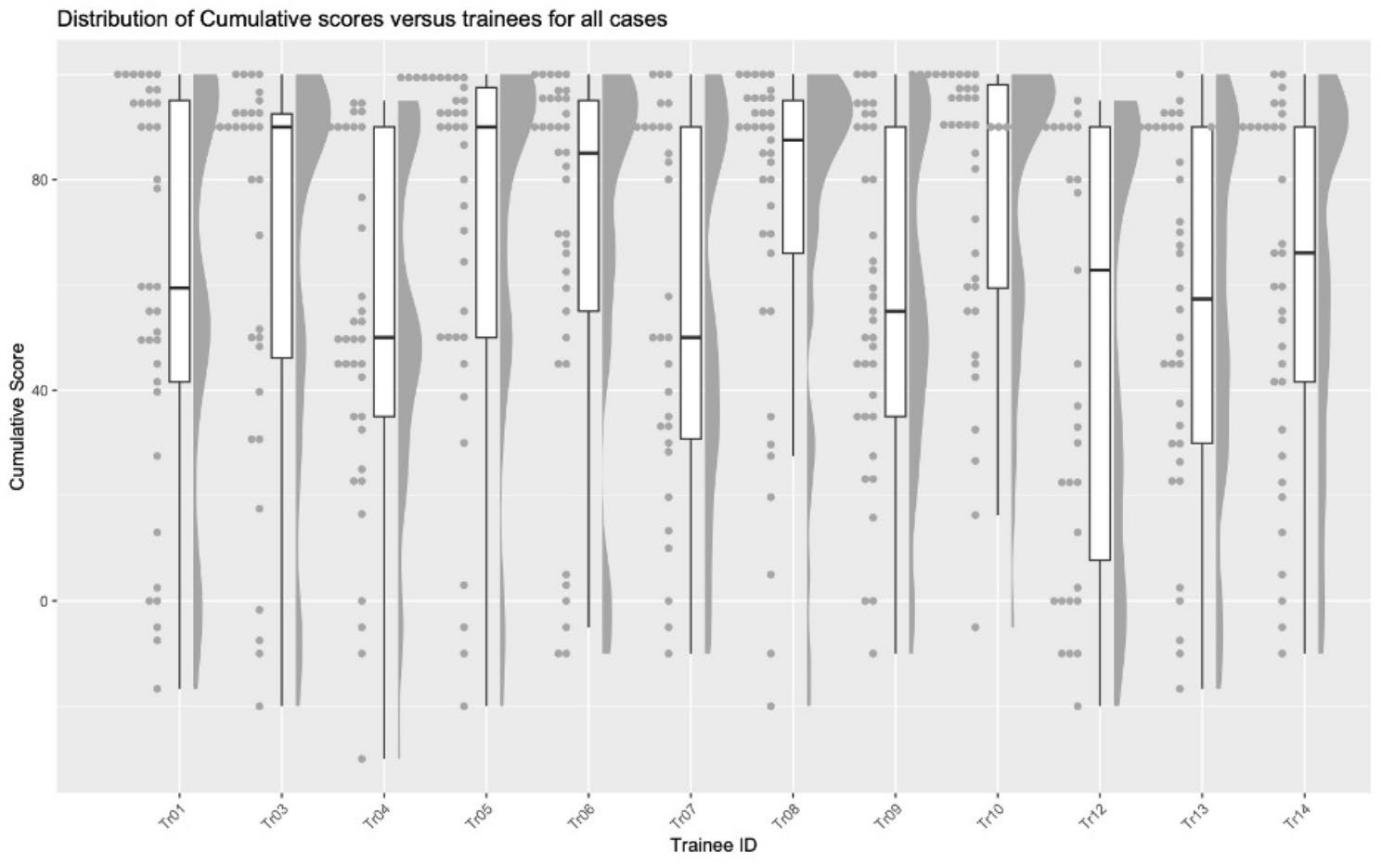
Raincloud plot showing the distribution of the cumulative scores and IQR by trainee

The cumulative scores for every case by trainee was calculated as described above. The distribution of these scores by trainee is shown in Fig. 5. The cumulative score ranged from − 30 to 100 with a mean of 61.9 ± 35.5 and median of 71.4 (IQR: 37.4, 92.0).

**Fig. 6:**
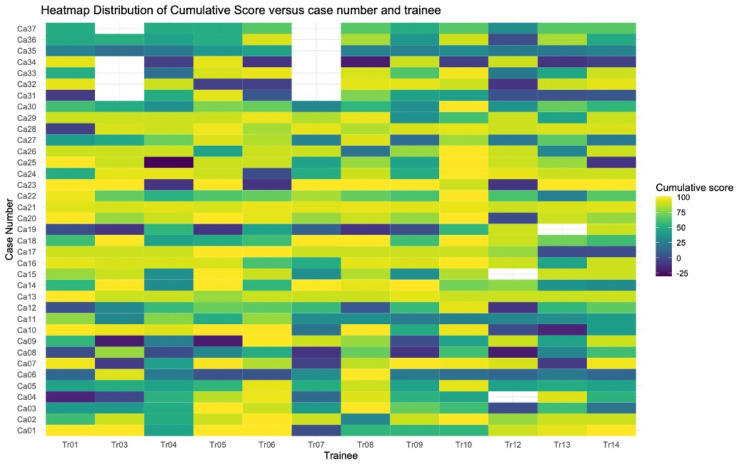
Heatmap for cumulative score by trainee for all cases

The heatmap in Fig. 6. shows the distribution of cumulative scores for all trainees by individual case number. Shades of colors range from dark blue (-30) to yellow (+ 100) depending on the cumulative score. A white cell corresponds to a case not interpreted by a trainee.

The cumulative scores on all the cases interpreted by the 12 trainees ranged from − 30 to 100. When averaging the cumulative scores of an individual trainee, three out of 12 trainees had a mean cumulative score > 70; four trainees had a mean cumulative score between 60 and 70, and four trainees had mean cumulative scores between 50 and 60. One trainee scored < 50. Similarly, the 3-score aggregate values across trainees showed that there were four trainees with average 3-score aggregates in each of the following ranges: 70–80, 60–70 and 50–60.

### Association between the scores and the length of training period of trainees

The overall mean acute diagnosis score across all cases was 0.68 ± 0.36. The simple linear regression that fitted a model with acute diagnosis score (continuous measure) as outcome against length of radiology training (categorical measure) as exposure, gave the following estimates: The mean acute diagnosis scores and SD by training period were 0.62 ± 0.03, 0.80 ± 0.05, 0.71 ± 0.05, 0.58 ± 0.07, and 0.66 ± 0.05 for trainees with ≤ 12 months, 12–24 months, 24–36 months, 36–48 months and > 48 months respectively. The mean acute diagnosis score of 12–24 months training was the only statistically significant greater score when compared to ≤ 12 months by the ANOVA with Tukey testing (*p* = 0.0002). We found a similar trend with distribution of 3-score aggregates and cumulative scores. There were no significant associations when the training period was categorized as less than and more than 2 years.

In our analysis of the number of overcalls versus length of training period, though there was a declining trend of the number of overcalls as the length of training period increased, it was not statistically significant.

### 3-score aggregate versus number of overcalls

**Fig. 7:**
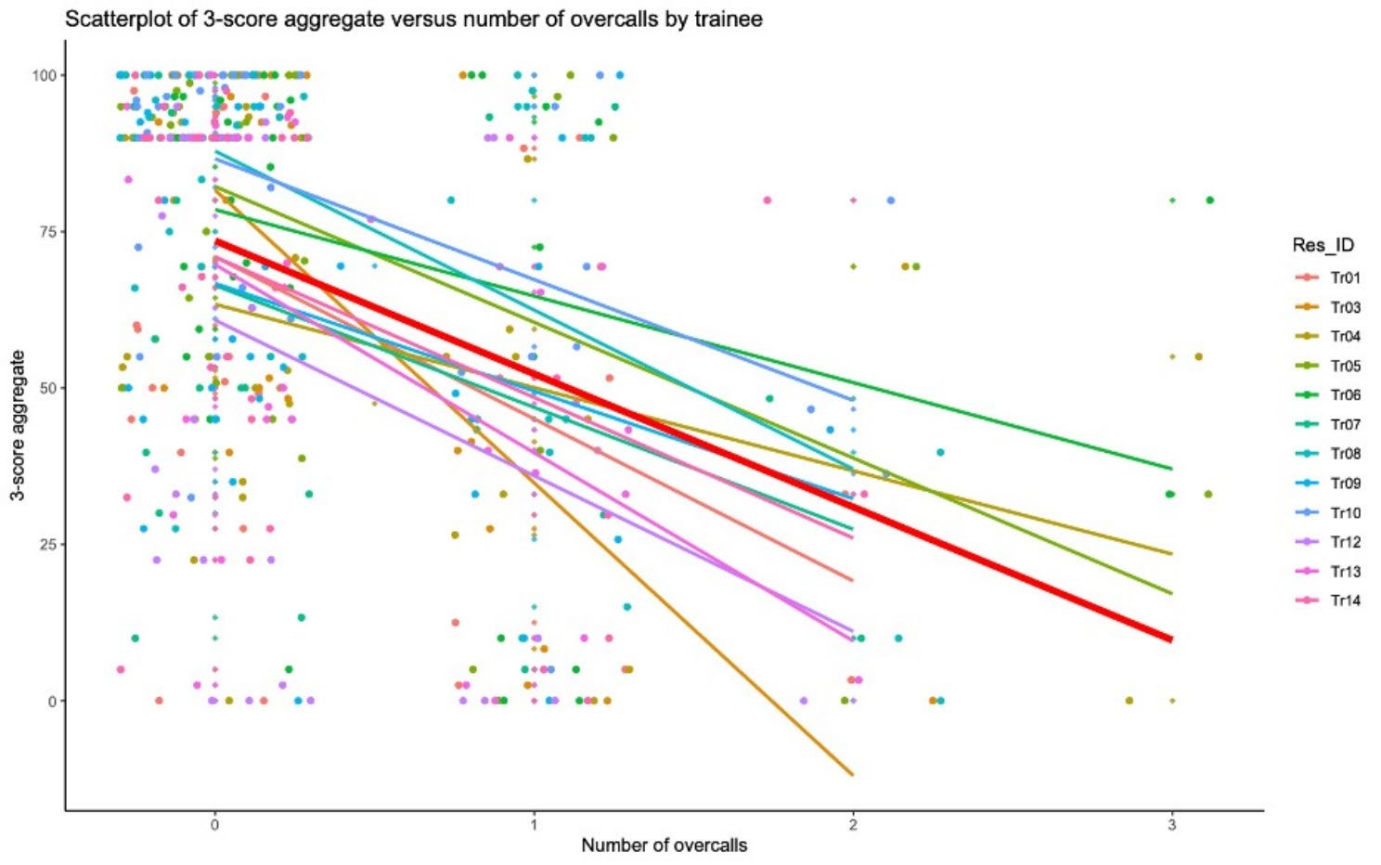
Scatter plot and trend line for 3-score aggregates versus number of overcalls for each trainee (overall group trend line in red)

We looked at the distribution of the 3-score aggregate versus the number of overcalls by trainee, and we found that the 3-score aggregate was inversely related to the number of overcalls. Results are depicted in Fig. 7, with the overall trend line represented in red.

## Discussion

Web-based radiology curriculum development has seen a broad expansion over the past years [10–12]. In contrast, there is an overall low number of radiology resident evaluation assessment tools as reviewed by Tu et al. [13]. More specifically, online radiology competency testing that accurately reproduces a true practice environment and evaluates trainees’ lesion detection and characterization skills on entire scans with actual radiology report generation has not been developed yet.

Objective structured clinical/practical examinations (OSCE/OSPE) have been widely used in medical education and have successfully integrated a single chest radiograph, or a few curated images for the purpose of evaluating medical students or radiography technologists [6–8]. However, OSCE/OSPE do not lend themselves to the evaluation of the actual practice of cross-sectional imaging (e.g.: formal CT or MRI scan interpretations) where a single clinical scan is made of hundreds of images that need to be thoroughly searched for abnormalities, then analyzed together to develop a final acute diagnosis. An OSCE approach based on a few curated images and focused questions would circumvent the searching task required of radiologists facing a clinical CT scan, a critical skill that needs to be assessed. Answering focused questions would fall short of generating a detailed radiology report with identification of a possible secondary diagnosis, incidental findings, and where possible errors in search and characterization would lead to the overcalling of normal findings.

In contrast, our aim was to test the ability of a radiologist trainee to systematically review and search through the hundreds of images of a clinical CT scan, identify abnormalities, and issue a formal scan interpretation as they would have to do on call in the hospital or later in the practice of radiology. That is what we refer to as an “authentic” setting because we were able to reproduce the exact conditions experienced by a trainee facing an entire scan on call. This departs from the standard board certification exams, PowerPoint-based testing, or OSCE/OSPE environment where providing only a few selected images of a CT scan and targeted questions leads the trainee directly to the most important images of a large image set thereby foregoing the task of systematically and methodically searching among hundreds of images, a core skill of the practice of radiology. Although this may be sufficient for testing medical students, it is not for evaluating radiology practitioners.

To our knowledge our study is the first demonstration of an on-line radiology testing tool developed to specifically enable the authentic review and interpretation of entire CT scans and their structured clinical reporting. Its strength lies in leveraging Lifetrack, an FDA-approved PACS that is run using Google Chrome from any laptop or desktop computer over an internet connection. No software installation or costly hardware is necessary, a unique advantage when developing a radiology testing approach for LMICs where resources are limited. Because it uses an FDA-approved PACS run over the internet, our approach inherently provides all the viewing tools usually available to radiologists when interpreting scans, from scrolling to windowing and measuring, all the way to multiplanar reformatting. There is also no limit to the number of images that can be submitted, and entire clinical CT scans were selected for the test we administered. Therefore, trainees reviewed entire CT scans in an authentic environment, searching for abnormalities and characterizing them, as if they were practicing radiology during an on-call shift, the ultimate test of a trainee’s abilities. Most published studies of online testing have relied on pre-selected sets of images from larger cross-sectional imaging studies or on the simpler task of assessing plain radiograph proficiency [14, 15] as have radiology and nuclear medicine board certification exams [16–18]. Moreover, these did not test for a trainee’s ability to search through a large volume of images to identify all abnormalities nor to determine whether these findings were interrelated.

Lifetrack provides a templated/structured reporting tool where trainees or practicing radiologists select short segments of sentences, key words, and modifiers from a comprehensive generic report template to compose grammatically correct sentences with great flexibility. As a result, the reports generated by the trainees follow a standardized and structured format enabling easy scoring and comparisons between trainees. It also enables the embedding of code in the active template software in order to perform automated exporting of answer data to a spreadsheet. In contrast most published studies of radiology testing tools and board certification exams have structured their answering process as a selection from a finite list of potential diagnoses that may hint at the area of interest in the images provided and that enables guessing [14–18].

We evaluated trainees for their ability to generate an accurate and complete clinical scan interpretation and presented a novel method to account for all the dimensions of such an interpretation. For each scan the trainee needed to identify all of the imaging features that contributed to the acute diagnosis responsible for the patient’s acute abdominal pain. In 11 cases the trainee was also expected to identify and characterize a second diagnosis unrelated to the acute abdominal pathology. In all 37 cases up to five incidental findings needed to be detected as well. For each of these three separate diagnostic tasks a score was attributed based on the report generated by the trainee with partial credit enabled. We combined the performances of a trainee in these three tasks by calculating a 3-score aggregate which reached 100% when the acute diagnosis, the secondary diagnosis (if present) and the incidental findings were all detected and properly characterized. A heavier weight was ascribed to the acute diagnosis score within the 3-score aggregate formula as it is the most critical task for acute patient care. In other words, the 3-score aggregate is designed to give a combined measure of the diagnostic skills of a trainee across all aspects of an accurate scan interpretation. Furthermore, when trainees misidentified a normal scan feature as abnormal or mischaracterized a truly abnormal finding, we graded this as an “overcall” as it could have deleterious effects on patient care through wrong treatments or unnecessary procedures being selected. Because such “overcalls” degrade the clinical value of the scan interpretation rendered, they were accounted for by subtracting from the 3-score aggregate resulting in the cumulative score defined above. We believe that our overall approach to scoring scan interpretations is novel and reflects more accurately all the skills required to perform an accurate scan interpretation. In contrast, previous studies graded trainees on the basis of answers selected from a finite list of potential diagnoses. Finlay et al. developed a web-based radiography test and proposed an “X-ray diagnostic accuracy scale” consisting of seven possible responses for continuous scoring of a trainee’s accuracy in interpreting an X-ray’s primary diagnosis [14]. Because a final report was not generated by trainees in these studies, they did not allow for the possibility of a concurrent unrelated diagnosis or incidental findings, nor for the evaluation of multiple imaging features of the acute diagnosis, or of overcalls.

We also introduced methods to analyze and display results and facilitate observations across cases and across trainees. With these displays one can easily detect patterns of interpretative difficulty in an individual trainee or for a specific pathology across multiple trainees. By re-administering this acute abdomen test with a different set of CT cases after a targeted educational intervention, these displays would help to visualize improvements in trainees. Targeted educational interventions can be delivered from remote using any on-line videoconferencing tool where the instructor shares the screen while scrolling through anonymized scans or while presenting didactic material all focused on a specific pathology. This teaching can be aimed at a group of residents or to an individual and does not require involving the limited educational resources of radiology departments in LMICs.

Our study was designed as a demonstration of a new on-line radiology testing tool and to share our experience in deploying it. It was not aimed at drawing analytical conclusions on a small sample. Within this limitation, our data clearly demonstrated a wide range of scores across 37 cases and 12 trainees allowing for several observations such as cases that were correctly interpreted by many trainees whereas others yielded low scores from most test takers. Because care had been taken to select CT cases that were representative of a pathology and not overly difficult, we believe that cases with low scores across multiple trainees reveal a need for further instruction of the trainees on that specific pathology. To reduce the potential bias of having experts select the cases, Boutis et al. reported methodology to select pediatric chest radiographs appropriate to evaluate the interpretive skills of medical students, with validation by pediatric trainees and emergency room physicians [15]. In contrast, our goal was to test the individuals ultimately responsible for the final interpretation of scans, namely radiology trainees taking on-call duties and radiology practitioners, thereby placing actual radiology practice expectations on the content of the scans and their difficulty. We mitigated the risk of undue difficulty in our test by having cases selected and vetted by both a junior radiologist practicing in Vietnam (TN) and a senior US academic radiologist (HV). Nonetheless, the analysis tools presented here enable the easy identification of cases that presented interpretation challenges for multiple trainees. Organizing our test material to contain multiple examples of an important pathology will enable a deeper evaluation of whether a specific example or a pathology are responsible for interpretive challenges. Finally, because our test requires the generation of an actual imaging report based on an extensive report template, it goes well beyond making a single final diagnosis and can identify which features of a scan are challenging. This versatility can be exploited for a finer analysis of trainee proficiency and suitability of the scans selected. Prior to using our test beyond educational observations and towards making formal competency-based evaluations of trainees, our proposed testing approach will require further validation according to the *Standards for Educational and Psychological Testing* [19] to ensure that we can trust the results of an assessment as emphasized by Tu et al. [13].

Contrary to our expectation and to the results reported by Finlay et al., we did not find a strong relationship between the various scores of a trainee and their length of radiology training [14]. This may be because of the small sample size of our study. In addition, as observed by most academic radiologists, trainees vary widely in their skills with some studying very regularly and interpreting as many cases as possible whereas others are less invested. After a year this can lead to significant gaps in expertise with some junior trainees outperforming more senior ones.

We found that the 3-score aggregate of a trainee is inversely related to their number of overcalls. This is in keeping with the expectation that trainees who overcall findings on a scan tend to be less accurate about the acute diagnosis, as well as the secondary diagnosis and incidental findings which are all included in the 3-score aggregate formula.

A limitation of our study rests with the scoring of scan interpretations which was performed by comparing structured text reports to a gold standard interpretation which is time consuming. We have successfully tested inserting code in the Lifetrack report template to extract acute diagnoses from individual reports and export them automatically to a spreadsheet. However, this only applied to the acute diagnosis and did not allow for partial credit yet as we have done with our manual scoring. The current report template does not enable the automatic extraction of the answers for the unrelated secondary diagnosis or incidental findings yet nor can it identify overcalls. Future goals will include leveraging the collaboration with Lifetrack Medical Systems to implement an automated scoring of scan interpretations.

Our first deployment of the Lifetrack-based online testing approach was successful. Care was taken to provide instruction to the trainees with respect to viewing scans with Lifetrack and using its active template reporting tool. This instruction and practice session was delivered to the trainees in Nairobi by a co-author (ES) from Manila (Philippines) in an online session using a few un-related CT scans of the Lifetrack teaching server. We also ensured that the computers all had Google Chrome installed and good internet access. All this was achieved remotely illustrating the power and flexibility of our Lifetrack testing approach. Each exam session allowed two hours to interpret six abnormal cases except for the last session which included an additional normal case. An average of 20 min per case was felt to be representative of the time afforded to a trainee during on-call duties to interpret a CT scan of the abdomen and pelvis. Each report generated with the Lifetrack active template has a timestamp associated with its release. From the session start time and the sequence of case report timestamps we can derive the duration of each scan interpretation which may provide insights on the trainees’ skills and on the complexity of each case.

The potential impact of the approach reported here is broad. Beyond the interpretation of CT scans for acute abdominal pain, our test could aim at the interpretation of CT for acute chest symptoms, headache, or trauma to name a few. Once a test of broad scope has been administered and its results analyzed, they can inform on targeted interventions needed to improve the level of proficiency of individual trainees or of the group. Our approach can equally test very focused or advanced concepts. The scans selected for a test will determine the focus, breadth and complexity of the knowledge being evaluated. Specifically, the test content should include pathologies unique to LMICs such as tuberculosis and parasitic diseases that are very uncommon in developed countries. This approach to testing radiology skills does not have to be limited to LMICs. It can be easily implemented by US diagnostic radiology or nuclear medicine residencies to test the proficiency of their trainees prior to having them assume on-call duties. It could be used to objectively gauge the proficiency of trainees at the end of each clinical rotation. This data could replace the recall-based summative evaluations known to be subjective and unreliable [20], and could avoid placing reluctant faculty in a position of having to provide negative feedback [21]. If certain interpretive skills are missing across most trainees, then the rotation content should be improved accordingly. Finally, because our methodology rests on anonymized actual scans, it can be deployed by any academic program and the test’s content can be renewed routinely so as to remain novel to the pool of trainees. Our Lifetrack-based radiology skills testing can be applied to any radiology modality from plain radiography to large cross-sectional image sets and even to videos of ultrasound exams. Consideration should be given to applying this technique to portions of board certification examinations. Because it tests trainees in an authentic fashion that reproduces a real radiology work environment, the rich data provided by our method can enable the study of radiology learning and the optimization of educational methods. It could as well be expanded to testing in disciplines relying heavily on imaging such as pathology, cardiology, dermatology, or ophthalmology.

By enabling an objective and authentic assessment of skills, followed by targeted educational interventions and reassessment, our Lifetrack-based approach can optimize the impact of limited local resources and of global health initiatives on the quality of radiology education in LMICs.

## Conclusions

We demonstrated the feasibility of remotely testing the authentic practice of radiology and showed that important observations can be made from our Lifetrack-based testing approach regarding radiology skills of an individual or a cohort. From observed weaknesses areas for targeted teaching can be implemented, and retesting could reveal their impact. This methodology can be customized to different LMIC environments and expanded to board certification examinations.

## Data Availability

The datasets used and/or analysed during the current study are available from the corresponding author on reasonable request.

## Abbreviations

LMICs: Low- and Middle-Income Countries
CT: Computed Tomography
PACS: Picture Archiving and Communication System
AKUHN: Aga Khan University Hospital in Nairobi
HICs: High-Income Countries
JCIA: Joint Commission International Accreditation
DJD: Degenerative Joint Disease
DVT: Deep vein thrombosis
TB: Tuberculosis
UVJ: Ureterovesical Junction
s/p: Status-post
IUD: Intrauterine Device
NG: Nasogastric
RLQ: Right Lower Quadrant
CBD: Common Bile Duct
GB: Gallbladder
IVC: Inferior vena cava
fx: Fracture

## References

[1] Omofoye TS. Radiology Education as a Global Health Service Vehicle. https://doi.org/101148/rycan220156 [Internet]. 2022 Nov 18 [cited 2023 Jul 14];4(6). 10.1148/rycan.220156 10.1148/rycan.220156 PMC9713591 36399040

[2] Rehani B, Brown I, Dandekar S, Sarkodie B, Mwango G, Rehani MM et al. Radiology Education in Africa: Analysis of Results From 13 African Countries. Journal of the American College of Radiology [Internet]. 2017 Feb 1 [cited 2023 Jul 14];14(2):247–52. http://www.jacr.org/article/S1546144016307554/fulltext 10.1016/j.jacr.2016.08.012 27818015

[3] Rehani B, Gao KT, Lau L, Rehani MM, Zhang YC, Dillon WP. Radiology Education in Asia: differences, similarities, and opportunities. J Am Coll Radiol. 2017;14(1):111–8. 28061957 10.1016/j.jacr.2016.08.013

[4] Iyawe EP, Idowu BM, Omoleye OJ. Radiology subspecialisation in Africa: A review of the current status. SA J Radiol [Internet]. 2021 [cited 2023 Jul 14];25(1). https://pubmed.ncbi.nlm.nih.gov/34522434/10.4102/sajr.v25i1.2168 PMC8424752 34522434

[5] Harden RM, Stevenson M, Downie WW, Wilson GM. Assessment of clinical competence using objective structured examination. Br Med J. 1975;1:447. 10.1136/bmj.1.5955.447. 1115966 10.1136/bmj.1.5955.447 PMC1672423

[6] Pérez Baena AV, Sendra Portero F. The objective structured clinical examination (OSCE): main aspects and the role of imaging. Radiologia. 2023;65:55–65. 36842786 10.1016/j.rx.2022.09.010

[7] Staziaki PV, Sarangi R, Parikh U, Brooks JG, LeBedis CA, Shaffer K. An objective structured clinical examination for Medical Student Radiology clerkships: Reproducibility Study. JMIR Med Educ. 2020;6(1):e15444. 10.2196/15444. PMID: 32374267; PMCID: 7240440. 32374267 10.2196/15444 PMC7240440

[8] Chew C, O’Dwyer PJ, Jaap A, McDowall S, Remers OJ, Williams J, McPhee I, Bjelogrlic P. Medical student assessments-frequency of radiological images used: a national study. BJR Open. 2020;2(1):20200047. 10.1259/bjro.20200047. PMID: 33367200. 10.1259/bjro.20200047 PMC7748984 33367200

[9] Jeffrey RB, Manaster BJ, Osborn AG, Rosado de Christenson ML. Diagnostic imaging. Emergency, 2007. ISBN-13: 978-1-4160-4934-0.

[10] Biswas SS, Biswas S, Awal SS, Goyal H. Current Status of Radiology Education Online: a Comprehensive Update. SN Compr Clin Med [Internet]. 2022 Aug 11 [cited 2023 Jul 14];4(1). https://www.researchgate.net/publication/362630778_Current_Status_of_Radiology_Education_Online_a_Comprehensive_Update 10.1007/s42399-022-01269-z PMC9366813 35971436

[11] Reid JR, Goske MJ, Hewson MG, Obuchowski N. Creating an International Comprehensive Web-Based Curriculum in Pediatric Radiology. American Journal of Roentgenology [Internet]. 2004 Nov 23 [cited 2023 Jul 14];182(3):797–801. https://www.ajronline.org/doi/10.2214/ajr.182.3.1820797 10.2214/ajr.182.3.1820797 14975987

[12] Omofoye TS, Leong LCH, Kalambo M, Teo SY, Lim WEH, Chew DCY, et al. Responsive web-based Breast Imaging Core Curriculum for International Radiology Residents with Self-Assessment: a pilot study. Acad Radiol. 2022;29(6):919–27. 34389260 10.1016/j.acra.2021.07.004 PMC9644401

[13] Tu W, Hibbert R, Kontolemos M, Dang W, Wood T, Verma R et al. Diagnostic Radiology Residency Assessment Tools: A Scoping Review. https://doi.org/101177/0846537120981581 [Internet]. 2021 Jan 5 [cited 2023 Jul 14];72(4):651–60. 10.1177/0846537120981581 33401932

[14] Finlay K, Norman G, Keane D, Stolberg H. A web-based test of residents’ skills in Diagnostic Radiology. Can Assoc Radiol J J Assoc Can Radiol. 2006;57:106–16. 16944684

[15] Boutis K, Pecaric M, Pusic M. Teaching X-ray interpretation: Selecting the radiographs by the target population. Med Educ [Internet]. 2009 May [cited 2023 Jul 14];43(5):434–41. https://www.researchgate.net/publication/24408510_Teaching_X-ray_interpretation_Selecting_the_radiographs_by_the_target_population 10.1111/j.1365-2923.2009.03311.x 19422490

[16] ABR [Internet]. [cited 2023 Jul 14]. https://www.theabr.org/diagnostic-radiology/initial-certification/core-exam

[17] ABR [Internet]. [cited 2023 Jul 14]. https://www.theabr.org/diagnostic-radiology/initial-certification/certifying-exam

[18] Sample Questions - ABNM [Internet]. [cited 2023 Jul 14]. https://www.abnm.org/exam/sample-examination-questions/

[19] American Educational Research Association., American Psychological Association., National Council on Measurement in Education. Joint Committee on Standards for Educational and Psychological Testing (U.S.). Standards for educational and psychological testing.:230.

[20] Neufeld VR, Norman GR. Assessing Clinical Competence: Springer Series on Medical Education (Volume 7). 1985.

[21] Collins J. Evaluation of residents, faculty, and program. Acad Radiol [Internet]. 2003 Feb 1 [cited 2023 Jul 14];10(SUPPL. 1):S35–43. http://www.academicradiology.org/article/S1076633203801484/fulltext 10.1016/s1076-6332(03)80148-4 12585442

